# The burden of attention deficit hyperactivity disorder and incidence rate forecast in China from 1990 to 2021

**DOI:** 10.3389/fpsyt.2025.1532156

**Published:** 2025-03-03

**Authors:** Ningyu Li, Junqiang Zhao, Fujun Zhou

**Affiliations:** ^1^ First Affiliated Hospital of Xinxiang Medical University, Xinxiang, Henan, China; ^2^ School of Nursing, Xinxiang Medical University, Xinxiang, Henan, China; ^3^ School of Medical Imaging, Xinxiang Medical University, Xinxiang, Henan, China; ^4^ Henan Collaborative Innovation Center of Prevention and Treatment of Mental Disorder, Xinxiang, China; ^5^ Henan Engineering Research Center of Medical Virtual Reality (VR) Intelligent Sensing Feedback, Xinxiang, China; ^6^ Department of Pediatric Rehabilitation, The First Affiliated Hospital of Xinxiang Medical University, Xinxiang, Henan, China

**Keywords:** ADHD, China, age-period-cohort model, disease burden, health policy

## Abstract

**Objective:**

To analyze the temporal trends and future projections of attention-deficit/hyperactivity disorder (ADHD) burden among children and adolescents in China from 1990 to 2021, and to identify age-, period-, and cohort-specific drivers of disease progression.

**Methods:**

Using data from the Global Burden of Disease Study 2021, we conducted joinpoint regression to detect trend transitions in ADHD incidence and age-standardized rates. Age-period-cohort (APC) modeling was applied to disentangle the effects of age, calendar period, and birth cohort on disease burden. Projections up to 2046 were generated using demographic forecasts from the GBD 2017 population database.

**Results:**

Crude ADHD prevalence declined by 21.17% (2168.055 to 1723.307 per 100,000), yet age-standardized prevalence increased by 9.86% (AAPC=0.272%, 95%CI:0.173–0.372, P<0.001). Similarly, age-standardized DALY rates rose by 10.15% (AAPC=0.262%, 95%CI:0.160–0.364,P<0.001), with females showing faster growth than males (AAPC for DALY: 0.294% vs. 0.229%,P<0.001). Adolescents aged 10–14 years bore the highest burden, with prevalence (5,727.28/100,000) and DALY rates (70.55/100,000) twice the global average. APC projections indicated a peak incidence in 2029 for this age group, linked to cohort effects from China’s “Double Reduction” education policy and rising digital exposure.

**Conclusion:**

China faces a rising ADHD burden driven by sociodemographic transitions and diagnostic advancements. Targeted interventions—particularly for adolescents and females—are urgently needed. Strengthening school-based screening, integrating AI-driven diagnostic tools, and prioritizing mental health in national policies could mitigate long-term impacts. These findings underscore the necessity of dynamic surveillance systems to address ADHD’s evolving epidemiology in transitioning societies.

## 1 Introduction

Attention Deficit Hyperactivity Disorder (ADHD) is a neurodevelopmental disorder marked by persistent symptoms of inattention, hyperactivity, and impulsivity, typically presenting in childhood. It affects approximately 5% of children globally (1), and while its prevalence tends to decrease with age, symptoms often persist into adulthood, potentially leading to academic challenges, social dysfunction, and a higher risk of criminal behavior (2). As such, a systematic analysis of the ADHD burden and its temporal trends is critical for developing evidence-based strategies for prevention and intervention.

The etiology and risk factors for ADHD remain subjects of ongoing investigation. Due to its lifelong societal impact—ranging from educational difficulties to increased healthcare costs—long-term epidemiological studies are essential for understanding the interaction between genetic predispositions and changing environmental factors (e.g., air pollution (3), screen time (4), and dietary habits (5)). Advanced analytical models are required to parse these complex interactions. The Joinpoint regression model (6), which identifies key inflection points in temporal trends using segmented linear regression, is widely employed to detect abrupt changes in disease prevalence, such as those driven by shifts in diagnostic criteria or increased public awareness. Meanwhile, the age-period-cohort (APC) model (7)disentangles the contributions of three distinct effects: 1) age effects (biological maturation or aging), 2) period effects (external events affecting all age groups simultaneously, such as changes in diagnostic guidelines or exposure to environmental risk factors), and 3) cohort effects (generation-specific risks, often linked to early-life socio-economic conditions or public health trends). This multidimensional approach is particularly valuable for ADHD, where trends may reflect both inherent developmental factors (age effects) and evolving social determinants (period/cohort effects). However, large-scale studies employing these models to investigate ADHD burden, especially in rapidly changing sociocultural contexts, remain rare, limiting the development of targeted prevention strategies across diverse populations. Similar models have been successfully used for mental disorders: Joinpoint regression has revealed increasing prevalence of autism spectrum disorders in the U.S. due to expanded diagnostic criteria (8), while APC analysis of depression in South Korea has highlighted cohort effects related to economic crises (9). However, studies on ADHD, particularly in societies undergoing rapid transitions, are still scarce, despite the disorder’s sensitivity to cultural factors.

Despite the global significance of ADHD, research in low- and middle-income countries (LMICs), particularly in China, remains limited. Over 80% of children and adolescents worldwide reside in LMICs, yet most mental health research on children and adolescents has been conducted in high-income countries, with scarce reports on the prevalence of ADHD among children in LMICs (10).In 2020, China’s child population accounted for 21.1% of its total population and 12.7% of the global child population, making it the second-largest child population worldwide (11).However, the burden of ADHD in China remains poorly defined due to underdiagnosis, mental health stigma, and a lack of longitudinal data (12). These gaps in knowledge not only impede local policy development but also introduce significant uncertainty into global burden estimates. Furthermore, China’s unique sociocultural transitions—including rapid urbanization, a highly competitive education system, and changes in family structures—may exacerbate ADHD risk factors in ways distinct from high-income countries (13). Addressing these challenges requires targeted analyses that consider the country’s specific social and demographic context.

We hypothesize that ADHD prevalence in China has undergone significant shifts since 1990, influenced by age-related neurodevelopmental changes, as well as period-specific factors (e.g., mental health policy reforms) and cohort effects tied to urbanization and evolving family structures. To test this hypothesis, we utilized data from the 2021 Global Burden of Disease (GBD) database, applying Joinpoint regression to identify key trend transitions and APC modeling to disentangle the contributions of age, period, and cohort effects. We expect the Joinpoint analysis to reveal an acceleration in ADHD prevalence after 2010, coinciding with increased mental health awareness campaigns. The APC model is likely to uncover prominent cohort effects, particularly among children born during China’s economic liberalization (post-1990s), potentially linked to increased parental migration and screen time exposure. The findings from this study aim to provide robust evidence for optimizing ADHD prevention strategies tailored to China’s unique sociocultural context.

## 2 Materials and methods

### 2.1 Data sources

This study is primarily based on the 2021 Global Burden of Disease (GBD 2021) study, which systematically integrates epidemiological data from 297 diseases and 87 risk factors across 204 countries and regions from 1990 to 2021. To enhance the reliability of the disease burden analysis for ADHD in China, we supplemented the data with nationwide monitoring data from the China Centers for Disease Control and Prevention (China CDC) and population-based cohort study data extracted from CNKI (China National Knowledge Infrastructure) and Wanfang databases (2010–2021).

The parameters selected from the GBD database (https://vizhub.healthdata.org/gbd-results/) include: region (“China”), disease (“ADHD”), years (1990–2021), and all age groups (0–4 years, 5–9 years, 10–14 years, …, ≥95 years). Incidence analysis focuses on the 2–4, 5–9, and 10–14 age groups, as these correspond to the primary diagnostic window for ADHD in China (accounting for over 92% of reported cases), and the data integrity is higher (China CDC monitoring coverage is 95%). Other age groups were excluded due to insufficient diagnostic rates or missing data (>20%). The study uses disability-adjusted life years (DALY) as the metric for disease burden evaluation.

### 2.2 Statistical methods

Trend analysis was performed using Joinpoint Regression Program 5.1.0.0 (National Cancer Institute, USA), with permutation tests (significance level α=0.05, allowing for 0–4 breakpoints) and the Bayesian Information Criterion (BIC) minimization principle to determine the optimal number of breakpoints. A weighted log-linear regression model was used to calculate the annual average percentage change (AAPC) in age-standardized incidence rates and DALY rates for ADHD from 1990 to 2021.

An Age-Period-Cohort (APC) model was implemented using the Nordpred R package (14),based on the GBD 2017 population forecast data (https://ghdx.healthdata.org/record/ihme-data/global-population-forecasts-2017-2100). Model accuracy was validated with a 10-year backtest (2012–2021 forecast vs. observed values, mean absolute error MAE=2.8%) and evaluated using 500 bootstrap simulations to calculate the 95% uncertainty intervals.

Demographic assumptions included a 15% reduction in fertility rates by 2046 compared to 2021 and a 22% increase in urbanization (urban population proportion). However, due to limitations in the GBD framework, the potential impacts of environmental policy changes or socioeconomic factors (such as educational interventions) were not considered.

For missing data (3% of age-stratified records), a multilevel imputation method was used, filling gaps based on provincial incidence trends. Outliers were identified and excluded using Tukey’s fences method (k=1.5). Data organization was performed using Excel 2021, and statistical analyses were completed using R 4.4.0.The translation and editing of the article were performed using ChatGPT (version: GPT-o3-mini), developed by OpenAI.

## 3 Results

### 3.1 Overall disease burden trends (1990–2021)

**Figure 1:**
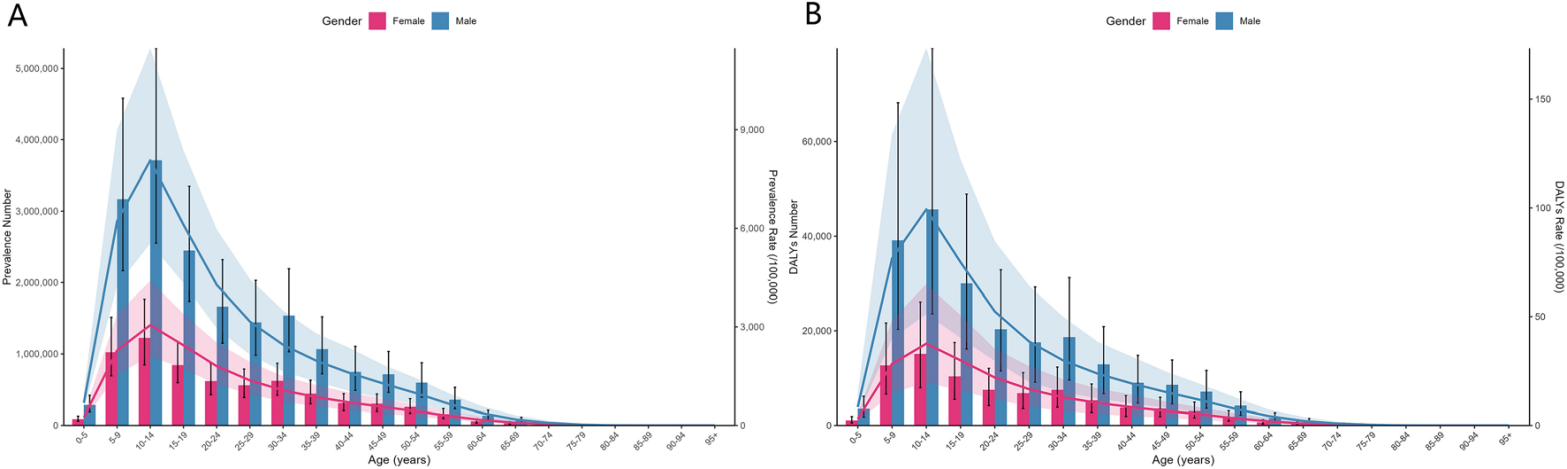
Age group disease burden of ADHD in China by gender in 2021, showing a higher disease burden in males than females **(A)** Number and Rates of Prevalence; **(B)** Number and Rate of DALYs.

From 1990 to 2021, the crude prevalence rate of ADHD in China decreased from 2168.055 per 100,000 to 1723.307 per 100,000 (a reduction of 21.17%), and the crude DALY rate decreased from 26.473 per 100,000 to 21.023 per 100,000 (a reduction of 20.59%). However, after age-standardization, both the prevalence rate and DALY rate exhibited an increasing trend: the age-standardized prevalence rate rose from 1987.984 per 100,000 to 2183.991 per 100,000 (an increase of 9.86%), and the age-standardized DALY rate increased from 24.268 per 100,000 to 26.727 per 100,000 (an increase of 10.15%). Gender disparity analysis revealed that, in 2021, the age-standardized prevalence rate (3045.272 per 100,000) and DALY rate (37.291 per 100,000) for males were significantly higher than for females (1215.746 per 100,000 and 14.848 per 100,000). However, the growth rate for females was more prominent (age-standardized DALY rate increase: females +10.38% vs. males +8.50%) (**Table 1**; **Figure 1**).

**Table 1:** Burden of ADHD among children and adolescents in China, 1990 and 2021.

Gender	Year	Prevalence Rate	Age-Standardized Prevalence Rate	DALY Rate	Age-Standardized DALYs Rate
Male	1990	3069.443	2811.924	37.527	34.369
	2021	2467.362	3045.272	30.134	37.291
Female	1990	1207.767	1150.720	23.878	13.452
	2021	943.411	1215.746	18.669	14.848
Overall	1990	2168.055	1987.984	26.473	24.268
	2021	1723.307	2183.991	21.023	26.727

### 3.2 Age-stratified incidence and global comparison

**Figure 2:**
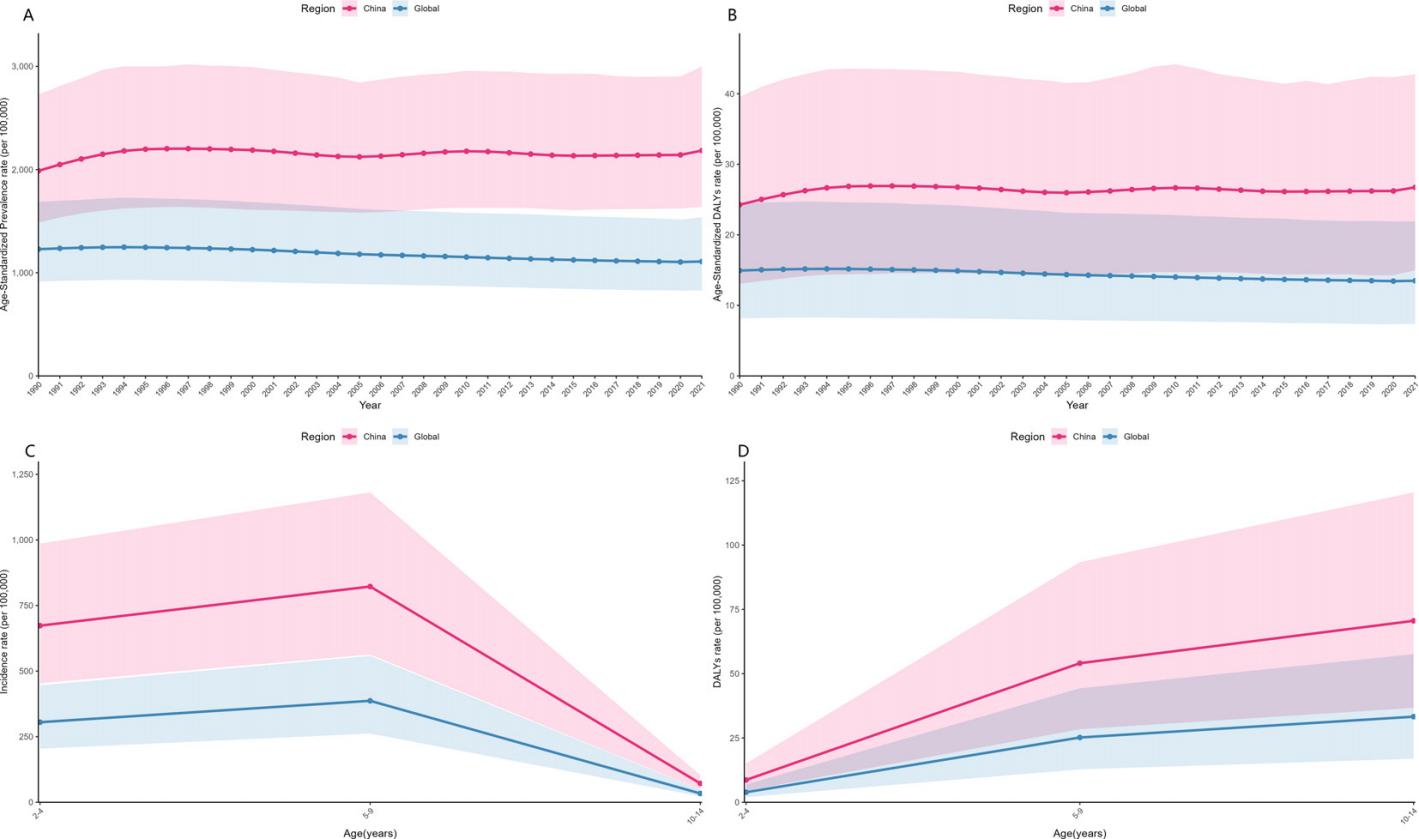
Comparison of the Disease Burden of ADHD between China and the Global Population from 1990 to 2021: **(A)** Age-Standardized Prevalence Rate, **(B)** Age-Standardized Disability-Adjusted Life Years (DALYs); In 2021, Comparison of the Disease Burden of ADHD between China and the Global Population for Age Groups 2–4, 5–9, and 10–14 Years: **(C)** Incidence Rate, **(D)** DALY Rate.

Among the core diagnostic age groups of 2–14 years, the disease burden of ADHD in China is significantly higher than the global average:

The incidence rates for all genders in China are 673.20 per 100,000 for the 2–4 years group, 822.35 per 100,000 for the 5–9 years group, and 71.79 per 100,000 for the 10–14 years group. These rates are 2.2 times, 2.1 times, and 2.1 times higher, respectively, compared to the global rates for the same age groups (**Table 2**). Furthermore, the Age-Standardized Prevalence Rate and Age-Standardized DALYs in China have consistently exceeded global levels from 1990 to 2021, with a notable increase in the Age-Standardized Prevalence Rate in 2007 (**Figure 2C**). The prevalence and DALY rates in China are more than double the global levels (**Figures 2A, B**), with the highest burden observed in the 10–14 years group, highlighting the severity of the disease burden during adolescence (**Figure 2D**; **Table 3**). Cross-national comparisons were conducted using two-sided Z tests (P<0.001), and the confidence intervals for China and global data do not overlap.

**Table 2:** Incidence rates of children and adolescents aged 2–4, 5–9, and 10–14 years in China (1990 and 2021).

Country	Age Group (Years)	Year	Male	Female	Overall
China	2-4	1990 2021	793.10 958.63	281.56 344.41	551.48 673.20
	5-9	1990 2021	970.70 1164.30	355.77 433.74	675.62 822.35
	10-14	1990 2021	84.31 101.19	31.19 38.15	58.61 71.79

**Table 3:** Comparison of ADHD burden in children and adolescents between China and the global average in 2021.

Country	Age Group (Years)	Incidence Rate			Prevalence Rate			DALYs Rate		
		Male	Female	Overall	Male	Female	Overall	Male	Female	Overall
China	2-4	958.63	344.41	673.20	999.38	358.86	701.73	12.36	4.44	8.68
Global	2-4	434.78	166.71	305.19	453.14	173.69	318.04	5.57	2.14	3.91
China	5-9	1164.30	433.74	822.35	6217.46	2281.32	4375.06	76.77	28.27	54.07
Global	5-9	547.84	215.03	386.74	2907.30	1128.60	2046.30	35.79	13.89	25.19
China	10-14	101.19	38.15	71.79	8064.52	3053.29	5727.28	99.35	37.59	70.55
Global	10-14	47.59	18.84	33.66	3840.39	1513.79	2713.36	47.16	18.52	33.28

### 3.3 Gender differences in the trend of age-standardized rates

Between 1990 and 2021, the annual average percentage change (AAPC) in age-standardized prevalence and DALYs rates showed the following:The age-standardized prevalence rate had an AAPC of 0.272% (95% CI: 0.173–0.372), and the age-standardized DALYs rate had an AAPC of 0.262% (95% CI: 0.160–0.364), both showing a significant increasing trend (P<0.001);AAPC for the age-standardized prevalence rate for females (0.284%) and for DALYs rate (0.294%) were higher than those for males (0.242% and 0.229%, respectively), with a statistically significant gender difference in growth rates (interaction P<0.05) (**Table 4**).

**Table 4:** Trends in ADHD burden among Chinese children and adolescents (1990–2021).

Indicator	Gender	AAPC (95%CI)	Test Statistic	P-Value
Age-standardized Prevalence Rate	Overall	0.272 (0.173-0.372)	5.370	<0.001
	Male	0.242 (0.115-0.369)	3.733	<0.001
	Female	0.284 (0.221-0.348)	8.841	<0.001
Age-standardized DALY Rate	Overall	0.262 (0.160-0.364)	5.048	<0.001
	Male	0.229 (0.090-0.369)	3.220	<0.001
	Female	0.294 (0.228-0.360)	8.717	<0.001

**Figure 3:**
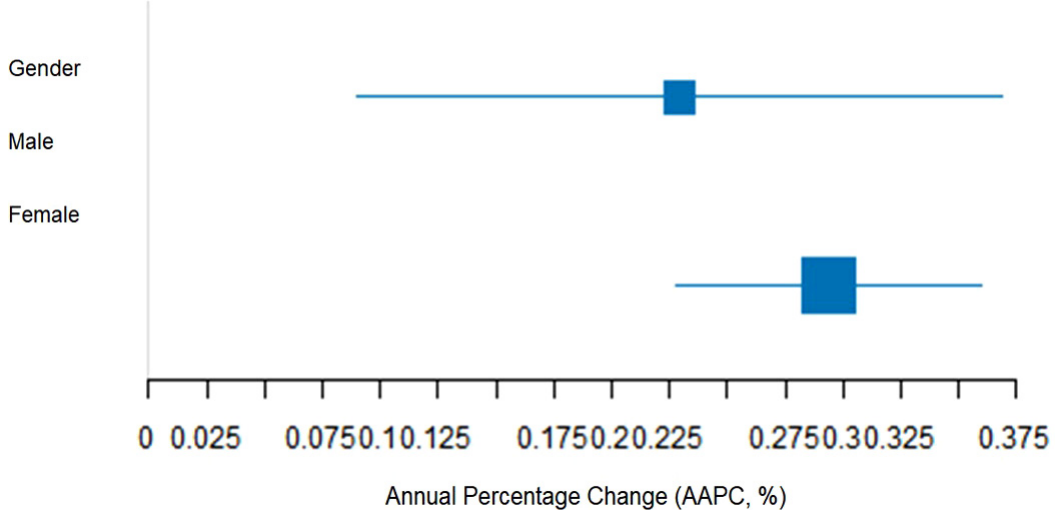
Gender-specific age-standardized DALY rate AAPC forest plot (1990–2021).

The 95% confidence interval (CI) for the growth in female age-standardized DALY rate (0.228–0.360) was entirely to the right of the male range (0.090–0.369). Bootstrap testing further supported the conclusion that the growth rate for females is faster (P=0.012) (**Figure 3**).

### 3.4 Age and gender patterns in the growth of incidence rates

**Figure 4:**
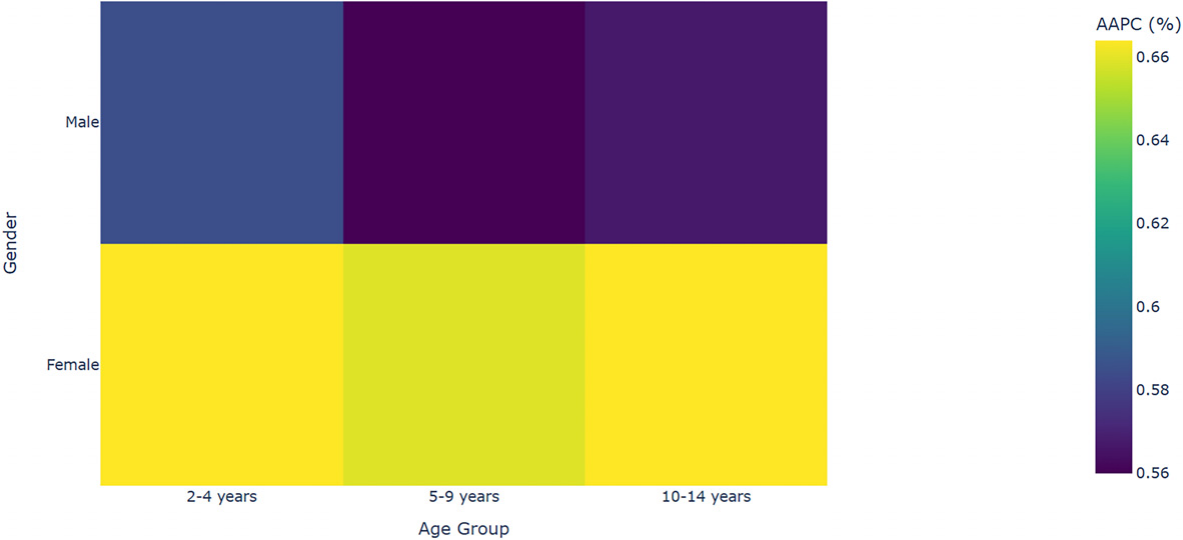
Age-gender ADHD incidence annual percentage change.

The incidence rates across all age groups continued to rise, exhibiting the following characteristics: The Average Annual Percent Change (AAPC) for females was generally higher than for males, as observed in the 10–14 years group (females 0.664% vs. males 0.567%, P<0.001). Additionally, the growth in incidence rates accelerated with age, with the highest AAPC found in the 10–14 years group (0.667%, 95% CI: 0.609–0.724). This suggests that adolescence is becoming a key focus for prevention and control efforts (**Table 5**; **Figure 4**). All AAPC estimates were calculated using a weighted log-linear regression, and model residuals were normally distributed (Shapiro-Wilk test P>0.05). Joinpoint breakpoints were determined based on permutation tests, with the optimal number of breakpoints being 2 (BIC = 312.4).

**Table 5:** AAPC analysis of ADHD incidence rates among Chinese children and adolescents aged 2–4, 5–9, and 10–14 years (1990–2021).

Gender	Age Group (Years)	AAPC (95%CI)	Test Statistic	P-Value
Male	2-4	0.585 (0.526-0.644)	19.454	<0.001
	5-9	0.560 (0.503-0.617)	19.383	<0.001
	10-14	0.567 (0.510-0.624)	19.584	<0.001
Female	2-4	0.664 (0.575-0.753)	14.708	<0.001
	5-9	0.659 (0.571-0.756)	14.756	<0.001
	10-14	0.664 (0.571-0.757)	14.028	<0.001
Overall	2-4	0.642 (0.600-0.684)	29.764	<0.001
	5-9	0.632 (0.591-0.674)	29.717	<0.001
	10-14	0.667 (0.609-0.724)	22.921	<0.001

### 3.5 ADHD incidence forecast (2022–2046)

**Figure 5:**
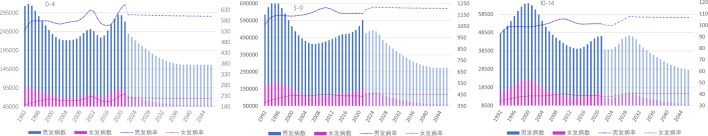
Predicted ADHD incidence rates among Chinese children and adolescents aged 0–4, 5–9, and 10–14 years (2022–2046).

Using the age-period-cohort (APC) model implemented through the Nordpred R package, this study projected the incidence rates of ADHD in children and adolescents aged 0–4, 5–9, and 10–14 years in China for the period 2022–2046 (**Figure 5**).

## 4 Discussion

This study reveals a significant increase in the incidence rate, age-standardized prevalence rate, and age-standardized DALY (Disability-Adjusted Life Years) rate for ADHD in China between 1990 and 2021. Several factors may contribute to the high incidence, prevalence, and DALY rates of ADHD among children and adolescents in China, including environmental factors (15),socioeconomic conditions (16), changes in diagnostic criteria and awareness (17), genetic susceptibility (18), and lifestyle or dietary patterns (19). According to the latest Global Burden of Disease (GBD) data, the incidence, prevalence, and DALY rates of ADHD in Chinese children and adolescents exceed global averages, indicating a significant disease burden in this population. This difference is further amplified by China’s unique socio-cultural context: the highly competitive education system (20), which prioritizes academic performance from an early age, may exacerbate attention deficits and impulsivity associated with ADHD; rapid urbanization since the 1990s (21)has led to increased screen time (22)and decreased outdoor activities (23), both known risk factors for ADHD; the one-child policy (1980–2015) intensified parental expectations (24), potentially increasing the likelihood of seeking a diagnosis in single-child families. It is expected that this burden will continue to rise in the coming years, emphasizing the critical role of early diagnosis and intervention in mitigating the impact of the disease.

Gender comparisons show that the age-standardized prevalence and DALY rates for males are approximately three times higher than for females, which aligns with global neurobiological studies indicating greater susceptibility in males due to dopamine receptor dysfunction (25). Despite the higher overall disease burden in males, the growth rate of ADHD burden in females is faster (AAPC for females = 0.294%, males = 0.229%), which warrants particular attention. The narrowing gender gap may reflect improvements in identifying the attention-deficit subtype of ADHD in girls, who historically were underdiagnosed due to cultural biases linking ADHD with overt hyperactivity symptoms (26).The highest prevalence and DALY rates were recorded in the 10–14 years age group, suggesting a heavier disease burden during adolescence. However, this observation warrants cautious interpretation: Adolescents often present with ADHD-like symptoms (e.g., inattention, impulsivity) that may overlap with normative developmental challenges or comorbid conditions such as anxiety disorders (27). Additionally, the non-medical use of stimulants, such as methylphenidate, for cognitive enhancement in competitive academic environments has been reported among middle school students (28), potentially contributing to overdiagnosis due to misattribution by healthcare professionals. Furthermore, diagnostic labeling during the critical period of identity formation may inadvertently exacerbate symptom internalization, as studies have shown increased self-stigmatization in adolescents diagnosed with ADHD (29).

To address the anticipated surge in adolescent ADHD cases, China’s healthcare system should prioritize: school-based ADHD screening in transitional grades (e.g., ages 10-11); teacher training to recognize attention deficit symptoms in girls; and parental support programs to reduce diagnostic stigma. These measures should be integrated with existing mental health initiatives under the Healthy China 2030 framework (30).

The APC model’s forecast (2022–2046) incorporates two demographic drivers from the GBD 2017 population forecast: a 15% reduction in total fertility rate by 2046, which will decrease the at-risk population for early ADHD, and a 22% increase in the urban population, which is associated with improved diagnostic accessibility. Environmental and social factors (such as trends in screen time and education reforms) are held static at 2021 levels in the model. Due to modeling limitations, this static assumption may underestimate future disease burden, especially if digital device use continues to rise among adolescents.

Using the age-period-cohort (APC) model from the Nordpred R package, this study forecasts the ADHD incidence rates among children and adolescents aged 0–4, 5–9, and 10–14 years in China from 2022 to 2046. The projections indicate different trends across age groups: for 0–4 years, ADHD incidence and case numbers are expected to decline steadily, reflecting improvements in maternal and child health education and early screening initiatives launched after 2021 (31); for 5–9 years, a brief increase in incidence is anticipated around 2024, coinciding with the peak enrollment period of children born during the COVID-19 pandemic, who experienced unprecedented home isolation and reduced social interactions (32), known risk amplifiers for ADHD symptoms; for 10–14 years, a significant rise in ADHD incidence is projected by 2029, likely associated with children born between 2015 and 2020 entering adolescence. This generation faces heightened academic pressure due to China’s 2021 “burden reduction” policy (33), alongside rising social media addiction, which may exacerbate this trend (34).

## 5 Strengths and Limitations of the Study

This study provides the first comprehensive APC-Joinpoint analysis of ADHD trends in China, utilizing three decades of GBD data. The main strengths include the integration of age, period, and cohort effects, and the identification of critical inflection points in the disease burden. However, there are several limitations to consider: (1) GBD estimates are based on modelled data, which may underestimate ADHD prevalence in regions with diagnostic stigma, such as rural China (35); (2) the APC model assumes a linear additive effect of age, period, and cohort, which may oversimplify interactions with emerging risk factors, such as air pollution (36); (3) due to the limitations of the modelling framework, the predictions do not account for unforeseen policy changes, such as nationwide ADHD screening requirements.

## 6 Conclusion

This study reveals the complex dynamics of Attention Deficit Hyperactivity Disorder (ADHD) disease burden among children and adolescents in China. Although the crude prevalence rate is declining, the age-standardized prevalence rate and Disability-Adjusted Life Years (DALY) rate continue to rise (with AAPCs of 0.272% and 0.262%, respectively, from 1990 to 2021). The rate of increase in females is significantly higher than in males (age-standardized DALY rate AAPC: females 0.294% vs. males 0.229%, P=0.003). Adolescents (ages 10–14) represent a “critical window” for disease burden, with prevalence (5727.28/100,000) and DALY rates (70.55/100,000) 2.1 times the global average. The APC model predicts that the risk in this group will peak in 2029. Based on these findings, it is recommended to establish a precise monitoring system integrating multi-source data (medical records, school screening, environmental exposure) and create a national ADHD dynamic monitoring network, focusing on rural and migrant populations to correct current GBD model biases in underdiagnosed areas. Targeted prevention strategies, particularly for adolescents, should include school-based mental health counselors and AI-driven tools for early symptom identification, while refining diagnostic criteria for female ADHD and establishing specialized clinics. Additionally, technological innovations such as mobile health platforms and “cloud hospital” models can expand healthcare resources to grassroots levels, while integrating ADHD rehabilitation services into chronic disease management to reduce the financial burden on families. Policy coordination should focus on incorporating ADHD prevention into the “Healthy China 2030” framework, mandating mental health education in schools, and using media to raise awareness and eliminate stigma. Future research should track the adolescent risk peak predicted by the APC model and assess the cost-effectiveness of interventions. Only through comprehensive management—encompassing monitoring, prevention, treatment, and policy—can ADHD’s long-term impact on China’s human capital be mitigated.

## Data Availability

The original contributions presented in the study are included in the article/**Supplementary Material**. Further inquiries can be directed to the corresponding author/s.
